# Health Technology Assessment in Osteoporosis

**DOI:** 10.1007/s00223-013-9724-8

**Published:** 2013-03-21

**Authors:** Mickael Hiligsmann, John A. Kanis, Juliet Compston, Cyrus Cooper, Bruno Flamion, Pierre Bergmann, Jean-Jacques Body, Steven Boonen, Olivier Bruyere, Jean-Pierre Devogelaer, Stefan Goemaere, Jean-Marc Kaufman, Serge Rozenberg, Jean-Yves Reginster

**Affiliations:** 1 Department of Health Services Research, School for Public Health and Primary Care (CAPHRI), Maastricht University, P.O. Box 616, Maastricht, 6200 MD The Netherlands; 2 Department of Public Health, Epidemiology and Health Economics, University of Liège, Liège, Belgium; 3 WHO Collaborating Centre for Metabolic Bone Diseases, University of Sheffield Medical School, Sheffield, UK; 4 Department of Medicine, Cambridge University Hospitals NHS Foundation Trust, Cambridge, UK; 5 MRC Lifecourse Epidemiology Unit, University of Southampton, Southampton, UK; 6 Physiology and Pharmacology Department, University of Namur, Namur, Belgium; 7 Department of Radioisotopes, CHU Brugmann, Université Libre de Bruxelles, Brussels, Belgium; 8 Department of Medicine, CHU Brugmann, Université Libre de Bruxelles, Brussels, Belgium; 9 Center for Metabolic Bone Diseases, Katholieke University Leuven, Leuven, Belgium; 10 Department of Rheumatology Saint Luc University Hospital, Université Catholique de Louvain, Brussels, Belgium; 11 Department of Rheumatology and Endocrinology, Ghent University Hospital, Ghent, Belgium; 12 Department of Endocrinology, Ghent University Hospital, Ghent, Belgium; 13 Department of Gynaecology–Obstetrics, CHU Saint-Pierre, Université Libre de Bruxelles, Brussels, Belgium

**Keywords:** Burden of disease, Cost-effectiveness, Economic evaluation, Health technology assessment, Osteoporosis

## Abstract

We review the various aspects of health technology assessment in osteoporosis, including epidemiology and burden of disease, and assessment of the cost-effectiveness of recent advances in the treatment of osteoporosis and the prevention of fracture, in the context of the allocation of health-care resources by decision makers in osteoporosis. This article was prepared on the basis of a symposium held by the Belgian Bone Club and the discussions surrounding that meeting and is based on a review and critical appraisal of the literature. Epidemiological studies confirm the immense burden of osteoporotic fractures for patients and society, with lifetime risks of any fracture of the hip, spine, and forearm of around 40 % for women and 13 % for men. The economic impact is also large; for example, Europe’s six largest countries spent €31 billion on osteoporotic fractures in 2010. Moreover, the burden is expected to increase in the future with demographic changes and increasing life expectancy. Recent advances in the management of osteoporosis include novel treatments, better fracture-risk assessment notably via fracture risk algorithms, and improved adherence to medication. Economic evaluation can inform decision makers in health care on the cost-effectiveness of the various interventions. Cost-effectiveness analyses suggest that the recent advances in the prevention and treatment of osteoporosis may constitute an efficient basis for the allocation of scarce health-care resources. In summary, health technology assessment is increasingly used in the field of osteoporosis and could be very useful to help decision makers efficiently allocate health-care resources.

Osteoporosis is a major cause of fracture worldwide, most notably of the hip, spine, and forearm. Osteoporotic fracture is strongly associated with morbidity, especially in terms of pain and disability. Hip and vertebral fractures are also associated with high mortality in the 2 years after the event [1, 2]. Osteoporosis is a common disease and is associated with a substantial health-care burden. In Western countries, one in two women and one in five men over the age of 50 years will experience an osteoporotic fracture during their remaining lifetime [3, 4]. Heterogeneity in hip-fracture risk is observed around the world [5], with estimates of a lifetime risk at the age of 50 years that vary from 1 % in women from Turkey to 28.5 % in women from Sweden [6]. The worldwide direct and indirect annual costs of hip fracture in 1990 were estimated at US$35 billion, with further increases predicted over the next 50 years [7]. In six major European countries, the burden of osteoporotic fractures was estimated in 2010 at €31 billion [8]. Fortunately, there is currently an array of diagnostic tools and effective treatments available for the management of osteoporosis [9].

Considering the limited health-care resources available, alongside major recent innovations in the management of osteoporosis, it is becoming increasingly important to allocate health-care resources appropriately and efficiently. Health technology assessment (HTA) aims to evaluate the clinical, economic, social, and ethical implications of the prevention and treatment of a condition—in this case, osteoporotic fracture—to guide national health-care policies (e.g., reimbursement decisions). The principal aim of HTA is to form a bridge between scientific experts in clinical practice and decision makers in health care in order to make the most appropriate use of available strategies for prevention and management. The ultimate target is evidence-based prioritization of national needs for health-care technology—be it for the prevention of fracture itself or management postfracture—for optimization of public-health initiatives. It was against this background that the Belgian Bone Club held a symposium to explore the issue from the clinician’s point of view. This article was prepared on the basis of the presentations and discussions surrounding that meeting, as well as review and critical appraisal of the literature. Our aim was to discuss the various aspects of HTA in osteoporosis, including epidemiology and estimation of the burden of disease, and to assess the cost-effectiveness of the recent advances in the management of osteoporosis.

## Health Technology Assessment

According to the International Network of Agencies for Health Technology Assessment [10], HTA is the systematic evaluation of “the medical, social, ethical and economic implications of development, diffusion, and use of health technology.” Its purpose is to support health-care decisions and inform policy making through objective information at the local, national, and international levels. The aim of HTA is to improve the quality of care by promoting an appropriate and rational use of health-care technologies [11] and by facilitating the introduction and dissemination of new technologies.

Health technology includes not only drugs, medical equipment, and devices but also prevention, diagnostic, and treatment procedures. HTA is conducted by interdisciplinary groups that use explicit analytical frameworks and draw from a variety of methods [10]. This field of research was developed in the 1970s and 1980s in the United States and Europe and has spread to the rest of the world over the last two decades [12]. HTA government agencies are now operating in many countries. They have been established to provide advice to governments and address, at the national level, the containment of health-care costs and the assessment of the impact of new technologies [13]. The organization of HTA and its influence on the public policy-making process can vary markedly between countries [14]. In addition, many research institutions are concerned with HTA [15], for example, the National Health Service Centre for Reviews and Dissemination in the United Kingdom. In 2012, the International Network of Agencies for Health Technology Assessment consisted of 53 members from 29 countries [10].

HTA is increasingly used by regulatory agencies to authorize a drug, device, or technology for market or reimbursement. HTA can be used to support decision making by clinicians and patients. It may also be used by other bodies, for example, associations of health professionals, hospitals (for acquisition of new technologies), and companies (to aid product development and marketing decisions) [16].

## Epidemiology and Burden of Osteoporosis

The first step of HTA is to assess the epidemiology and burden of the disease or outcome concerned. Epidemiological studies performed in the early 1990s in white North American individuals aged over 50 years indicated that the lifetime risk for any fracture of the hip, spine, or forearm was 40 % in women and 13 % in men [17]. Similar rates of fracture were reported in a study performed 10 years later in the UK General Practice Research Database, with values of 53 % for women and 21 % for men [18]. These data include fractures not linked to osteoporosis, such as those of the skull, hands or fingers, and ankles or toes. Lifetime risk for fracture of the hip, spine, and wrist has been estimated as 14, 28, and 13 %, respectively, for women in the United Kingdom and 3, 6, and 2 % for their male counterparts [7]. The risk of fracture rises progressively from the age of 50 years, and there is a substantial female excess at all time points above that age.

Fracture rates are known to vary considerably according to geographical location [5], which also influences HTA. Age-standardized incidences of hip fractures are currently available in 63 countries [5]. Age-standardized incidence rates of hip fracture in Europe and North America are generally higher than those in Asia and Africa, and there is also a large difference within Europe (763 per 100,000 women in Norway vs. 418 per 100,000 women in England) [19]. These differences correlate weakly with latitude [20], activity [21], and fall risk [19, 22] but not with bone mineral density (BMD). Geographical differences may be partly explained by time trends. Age, period, and birth cohort all impact on secular trends in hip fracture [23, 24], suggesting that there are determinants that operate throughout life; for example, even maternal vitamin D status may play a role [25].

Data are available regarding incident trends in hip fracture from around 1928 up to the present. Steep and statistically significant increases in age-adjusted rates among men and women were observed in the middle to late twentieth century. However, while global projections for hip fracture in the 1990s suggested sustained increases due to demographic changes in populations [26], there is evidence that the trends in incidence are reaching a plateau or may even have declined. This trend is most consistent in the United States, where hip-fracture rates and subsequent mortality are declining (though with coincident increase in morbidities associated with hip fracture) [27]. There is also evidence for similar trends in Europe and Oceania but not (for the time being) in Asia [28, 29]. In Belgium, the age-standardized incidence of hip fracture fell from 5.60 per 1,000 women aged over 50 years in 2000 to 5.22 per 1,000 in 2007 [30]. These data (excluding readmissions) also highlight a reversal of the secular trend for hip fracture in Belgian women, with a 1.1 % reduction in the average yearly change in the incidence of hip fractures in the period 2000–2007 [30] compared with a 2.1 % increase reported between 1984 and 1996 [31]. The reasons for this reversal are not entirely clear, though it could be linked to changes in risk factors [28], most notably those acting in later life; for example, changes in patterns of physical activity, vitamin D insufficiency, and increasing survival of the frailest elderly were likely to contribute to the rise in hip-fracture incidence in the second half of the century. On the other hand, reduction in rates of hip fracture in the last two decades may be linked to wider use of osteoporosis treatments—and some studies have revealed that the recent decrease in hip-fracture incidence coincided with increased use of osteoporosis treatments [27, 30, 32]—as well as other possible factors, such as increased rates of obesity or improvements in nutrition or tobacco consumption. However, there is no single explanation, and no causal relationship can be ascertained between the increase in the use of osteoporosis medications and the decrease in hip-fracture incidence [30, 33]. Further research is necessary to explore these trends in more depth. Despite a reduction in age-adjusted incidence in many countries, the absolute number of fractures is still increasing due to the aging of the population and increasing life expectancies. In Belgium, for example, the absolute number of hip fractures increased by 9 % between 2000 and 2007 [30].

A report launched by the International Osteoporosis Foundation in collaboration with the European Federation of Pharmaceutical Industry Associations has revealed the immense burden of osteoporotic fracture [8]. For the year 2010, approximately 2.5 million new fractures occurred in Europe’s five largest countries (France, Germany, Italy, Spain, United Kingdom) and Sweden alone [8]. The economic impact of these fractures was estimated to be nearly €31 billion in that year [8]. Approximately 34,000 deaths were causally related to these fractures, and the burden expressed in quality-adjusted life years (QALYs) was estimated at 850,000 QALYs. Considering current trends in demography, the burden of osteoporosis is expected to further increase in the near future. The projected number of fractures in these major countries is 3.2 million by 2025, an increase of 29 % [8].

## Recent Advances in the Treatment of Osteoporosis

The diagnosis and treatment of osteoporosis are rapidly evolving. A variety of new treatments for osteoporosis have become available over the past few years [34]. Fracture risk assessment is increasingly used to guide treatment decisions [35], and the impact of nonadherence with osteoporosis medications on treatment efficacy has led to the development of behavioral interventions to improve adherence [36, 37]. Assessment of these major advances from a clinician’s point of view is provided below, while the economic assessment will be discussed later.

### Novel Treatment Strategies

Over recent years, new treatment strategies have become available to prevent and treat osteoporosis, including bazedoxifene [38], denosumab [39], ibandronate [40], strontium ranelate [41], and zoledronic acid [42]. Other promising drugs are currently in development, such as odanacatib (a specific inhibitor of the osteoclast protease cathepsin K) and antibodies against the sclerostin and dickkopf-1 proteins [34]. Systematic review of the clinical efficacy, effectiveness, and side-effect profiles of these drugs is a crucial part of HTA. Good-quality systematic reviews of the evidence for the efficacy and safety of these drugs are available [9, 34, 43–46] and will not be discussed further here.

### Fracture Risk Assessment

Evaluation of risk and prediction of outcome is another important component of HTA. It is well established that BMD is inversely related to fracture risk [47]. For every 1.0 SD decrease in BMD at the hip, spine, or radius, there is an approximately 1.5- to 2-fold increase in fracture risk at any site. Measurement of BMD is therefore an integral part of the prediction of fracture risk. However, there are a host of other clinical risk factors that can improve fracture risk prediction, notably because they increase fracture risk in a manner that is at least partially independent of BMD. Examples are a prior history of fragility fracture, a parental history of hip fracture, current smoking, high alcohol intake, systemic glucocorticoids, and the presence of rheumatoid arthritis [48]. Fracture risk-prediction algorithms have been generated to combine results of BMD assessment with the presence of clinical risk factors, thereby improving the prediction of osteoporotic fracture.

Current algorithms generally produce estimates of 10-year risk of fracture. The most widely used is the World Health Organization (WHO) fracture risk-assessment tool, FRAX^®^, which is recommended by guidelines in North America, Europe, and Japan. The FRAX algorithm was developed using international population-based data for men and women aged 40–90 years. FRAX combines 11 parameters of risk (femoral neck BMD, age, sex, body mass index, prior fracture, parental history of hip fracture, rheumatoid arthritis, glucocorticoids, smoking, alcohol, and secondary osteoporosis) to calculate a 10-year probability for major osteoporotic fracture and for hip fracture [35]. Other fracture risk-prediction algorithms have also been produced, which are not based on probability (i.e., do not incorporate the death risk) and are less widely used [49–51]. A simpler score, produced by Ensrud et al. [49], used a United States-based population of women aged 65 years or above to determine a 10-year risk of major osteoporotic or hip fracture using the risk factors of age and previous fractures with and without BMD. They considered that this simpler model may predict risk as well as the more complex FRAX algorithm, but this is the subject of some debate [52]. The Garvan Fracture Risk Calculator includes BMD, age, sex, previous fracture, and falls to produce 5- and 10-year risks of any fracture in men and women aged over 60 years [51]. Finally, the QFracture algorithm employs multiple risk factors, including comorbidities, medications, and falls, but not a prior fracture or BMD to estimate 2-, 5-, and 10-year risks of hip, wrist, and vertebral fracture [50].

The FRAX algorithm is the most widely used tool and has been endorsed by international guidelines. However, it does have a number of limitations; for example, it only allows for inclusion of femoral neck BMD but not BMD values at other sites. Moreover, FRAX does not incorporate the notion of dose response for some of the risk factors, for example, previous fracture and glucocorticoids [53]. Simple guidance for the adjustment of fracture probabilities on the basis of exposure to glucocorticoids and information on lumbar BMD are available [54, 55]. FRAX, like all the models except QFracture (which ignores all previous fracture), may also underestimate risk if previous vertebral fractures are not accounted for, despite established evidence for the influence of incident fracture. Moreover, it does not formally take into account the number of previous fractures. The recent observational cohort Global Longitudinal Study of Osteoporosis in Women (GLOW) collected information on 50,000 women in 10 countries [56]. Compared to women with no previous fracture, the hazard ratio for incident fracture was 1.81 (95 % confidence interval [CI] 1.66–1.97) in patients with one prior fracture, 2.98 (95 % CI 2.63–3.38) for those with two prior fractures, and 4.80 (95 % CI 4.11–5.60) for those with three prior fractures [56]. Similarly, the presence of undiagnosed vertebral fracture was associated with a substantially increased risk for hip and new vertebral fracture [57] but could only be incorporated in risk-prediction algorithms by systematic evaluation of spinal radiographs. Clearly, this is not feasible for all consultations, though possible indications for vertebral imaging in fracture assessment should include low BMD, height loss, kyphosis, pain suggestive of a vertebral fracture, previous nonvertebral fracture, and reduced rib-to-pelvis distance. One potential drawback to FRAX may be that it does not include falls, which clearly contribute to the occurrence of fracture and are included in other risk tools [50, 51]. Although there is some evidence that including falls in FRAX would improve fracture-risk prediction [58], it may be problematic for a number of reasons, discussed elsewhere [53].

In conclusion, FRAX and other fracture-risk algorithms enable fracture prediction based on clinical risk factors with or without BMD and provide a basis for setting intervention thresholds. Current strategies for external validation and comparisons of fracture-risk algorithms involve procedures of discrimination, calibration, classification, and decision curve analysis, all of which have drawbacks and require further study [52].

### Adherence to Treatment

The problem of medication nonadherence has emerged as a critical hurdle to osteoporosis management. Adherence with osteoporosis medications is poor and suboptimal [59–61]. Several studies have suggested that between 50 % and 75 % of women who initiate oral bisphosphonate therapy are nonadherent within 1 year. Poor adherence reduces the effectiveness of osteoporosis treatment, resulting in lower BMD gains and subsequently higher fracture rates [62, 63]. Approximately 50 % of the potential clinical benefits of oral bisphosphonates are lost due to nonadherence [36, 37, 64], and the costs per QALY from these medications are doubled when assuming nonadherence [64]. Nonpersistence is the leading problem, causing more than 90 % of the clinical and economic burden of poor adherence [64].

Over the past few years, behavioral interventions and treatments with longer intervals between doses have been developed in order to improve medication adherence. Systematic reviews of these interventions have identified a limited number of studies of variable quality suggesting that some intervention techniques may help to improve medication adherence, but this requires further investigation [65, 66]. Different dosing regimens [67], the use of a decision aid [68], and education programs [69] may also improve medication adherence.

## Economic Evaluation

Economic evaluation is as important a branch of HTA as the epidemiological and treatment aspects. The aim of economic evaluation is to examine the outcomes and costs of health-care interventions; it could be defined as the comparative analysis of two or more health-care interventions in terms of both costs and impact on outcomes [70]. By informing decision makers about the relative cost-effectiveness of different health-care interventions, economic evaluation can help decision makers to make rational decisions and efficiently allocate resources. Cost-effectiveness is currently considered to be the fourth hurdle in drug development, behind quality, safety, and efficacy [71]. Although the most common application of economic evaluation is drug pricing and reimbursement [72], the implementation and viability of any other health intervention (such as screening or information campaigns) also depend on their evaluation and relative cost-effectiveness.

With the rising demand for health care, budget constraints, and the rapid development of health technologies, economic evaluation plays an increasingly large role in the decision-making process for health-care interventions. This has led to an increase in the number of published economic evaluations in the literature and to an increased use of economic data in the health-care decision-making process (in particular for drug reimbursement). Many countries currently require economic evaluation as part of the reimbursement process for drugs [73].

The four main types of economic evaluation all approach costs in the same way but differ in the way they approach outcomes [70]:

- *Cost*-*minimization analysis* is used where the consequences of two or more interventions are broadly equivalent, so the difference between them is limited to a cost comparison. This approach is only meaningful for agents with similar efficacies or side effects, which is difficult to apply to a heterogeneous class like the osteoporosis drugs [74].
- *Cost*-*benefit analysis* measures both costs and benefits in monetary terms. This approach aims to demonstrate that a program will yield to a net welfare gain and ranks interventions according to the net benefit they provide. The practical difficulties of measurement and valuing health benefits have limited the use of this type of analysis in health care [75].
- *Cost-effectiveness analysis* compares costs and outcomes expressed in a single dimension, such as fractures saved, BMD gained, or life-years gained.
- *Cost*-*utility analysis* is considered to be a specific case of cost-effectiveness analysis where the outcome measure is expressed in QALYs. The QALY estimator is an attractive outcome measurement in the field of osteoporosis because it offers the advantage of simultaneously capturing the benefits from a reduction in mortality and from a reduction in morbidity [76]. In addition, this approach allows comparison across different health programs and diseases using a generic unit of measure.

There are different categories of costs that may or may not be included in an economic evaluation. It is essential to specify and justify the perspective in which the analysis is undertaken. The most common perspectives used are those of health-care payers and society. The societal perspective is the broadest, including direct and indirect medical costs, and is theoretically preferred [70]. However, most local guidelines recommend the use of a health-care payer perspective [73].

**Fig. 1:**
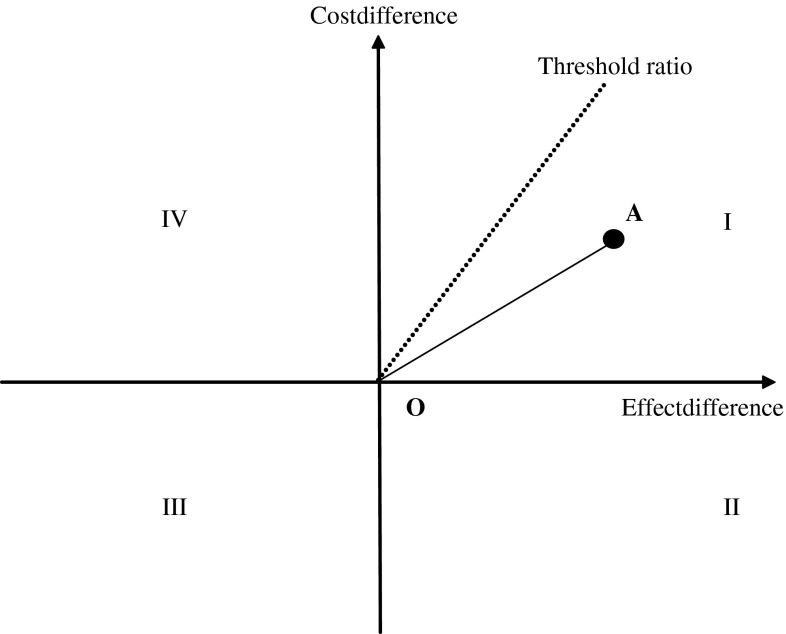
Cost-effectiveness plane. The difference in quality-adjusted life-years between intervention *A* and comparator *O* is represented on the horizontal axis and the difference in cost, on the vertical axis. The slope of the line between intervention *A* and comparator *O* is the incremental cost-effectiveness ratio. If *A* is located in quadrant II or IV, it is dominant (more effective and less costly than comparator O); in quadrant IV, intervention *A* is less effective and more costly than *O*. In quadrant I, *A* is more effective but more costly; and in III, it is less effective and less costly. The choice will depend on the cost-effectiveness threshold that represents the maximum amount the decision maker is willing to pay for a unit of effectiveness. Interventions that fall below the cost-effective threshold would be deemed cost-effective

The results of a cost-effectiveness analysis or cost-utility analysis are usually expressed in terms of the incremental cost-effectiveness ratio (ICER), which is defined as the difference in terms of costs between two interventions divided by their difference in effectiveness. An ICER represents the additional cost of an intervention per effectiveness unit (e.g., fracture saved or QALY gained) versus the comparator. The results can be presented graphically on the cost-effectiveness plane (Fig. 1), where the difference in effectiveness between intervention A and comparator O is represented on the horizontal axis and the difference in cost, on the vertical axis [77]. If A is located in quadrant II or IV, the choice is straightforward: in quadrant II, intervention A is more effective and less costly than comparator O and said to be dominant; in quadrant IV, intervention A is less effective and more costly than O and should be rejected. In quadrants I and III, there is no obvious decision: intervention A is either more effective and more costly than comparator O (quadrant I) or less effective and less costly (quadrant III). The choice will depend on the maximal amount the decision maker is willing to pay (or accept) for a unit of effect (e.g., a fracture prevented or a QALY). The slope of the line between intervention A and comparator O is the ICER. As shown in Fig. 1, if intervention A falls below the ICER threshold, then it is deemed cost-effective.

In order to draw conclusions about an intervention’s cost-effectiveness, the ICER should be compared with a cost-effectiveness threshold, above which the intervention would be deemed not cost-effective (because the additional cost for an additional unit of effect is too high) and below which it would be deemed cost-effective. The United Kingdom currently uses a threshold of £20,000–30,000 per QALY gained [78], though most other countries define no generally accepted or recommended thresholds for cost-effectiveness. The objections to the specifications of a fixed cost-effectiveness threshold are numerous. First, any threshold for cost-effectiveness would be somewhat arbitrary and variable over time. A threshold would also vary between countries to reflect differences in resources. The WHO has suggested a cost-effectiveness threshold based on evaluating each disability-adjusted life-year as three times the gross domestic product (GDP) per capita [79]. On this basis, a willingness-to-pay of two times GDP per capita was used to define intervention thresholds in osteoporosis [80, 81]. In addition, health-care decision making remains a multifactorial process and depends on many factors other than cost-effectiveness. As decisions are not solely based on the ICER, it is probably not necessary to define a fixed threshold below which an intervention can be considered cost-effective. This should, however, not be used as an argument against the use of economic considerations in health care [82]. In most countries, interventions with a low ICER have a higher probability of being adopted/accepted than those with a high value [82, 83]. Factors to consider alongside cost-effectiveness include burden of disease, uncertainty regarding cost-effectiveness, lack (or inadequacy) of alternative treatments, and overall financial implications for government [84]; the seriousness of the disease and equity objectives are also important. Recently, the UK National Institute for Health and Clinical Excellence (NICE) introduced new criteria and increased the threshold for end-of-life treatments [85].

Economic evaluation can be performed alongside randomized, controlled trials [86] or separately using decision-analytic modeling [87]. The first approach estimates costs, effects, and utilities using individual patient data [88] but suffers from a number of limitations that reduce its usefulness in informing decision makers about the economic value of interventions. These include, for example, a failure to compare with all relevant options, a truncated time horizon, and a lack of relevance of the decision context [89]. In addition, reliance on a single trial may ignore results from other clinical trials, meta-analyses, and observational studies [87]. Decision-analytic models are therefore becoming a necessary feature for estimating the economic value of health interventions. This is especially true in osteoporosis since the prevention of an osteoporotic fracture (in particular of the hip or vertebra) has long-term consequences on costs and outcomes that may not be captured by trial data.

Health-care modeling involves the application of mathematical techniques to summarize available information about health-care processes and their implications [90], usually with computer software. A model aims to represent the complexity of the process in a simple and comprehensible form [91]. Modeling is useful to extrapolate beyond clinical trials; to combine multiple sources of evidence; to incorporate epidemiological, clinical, and economic data; and therefore to answer more relevant policy questions [90]. In addition, modeling is appropriate at the early stages of the development of a new technology to inform research priorities prior to initiation of clinical trials [90, 91].

There may be some problems with using modeling in the economic evaluation of health care [92]. Inappropriate use of modeling could lead to unreliable conclusions, as would be the case for combination of evidence from incompatible studies with a high degree of uncertainty and oversimplification of some aspects of reality [88, 90]. Manipulation could also be greater when modeling reflects commercial and government interests [93]. An example is the discussion about the appraisal of NICE on the health economic assessment of interventions for the primary and secondary prevention of osteoporotic fractures in postmenopausal women in the United Kingdom [94]. Some authors do not support the view of the NICE guideline and doubt the validity of the model and the appropriateness of its use to inform guidance [95]. Interestingly, a recent study has shown that funding source (industry vs. nonindustry) did not seem to significantly affect the reporting of low or high ICERs for bisphosphonates [96].

**Fig. 2:**
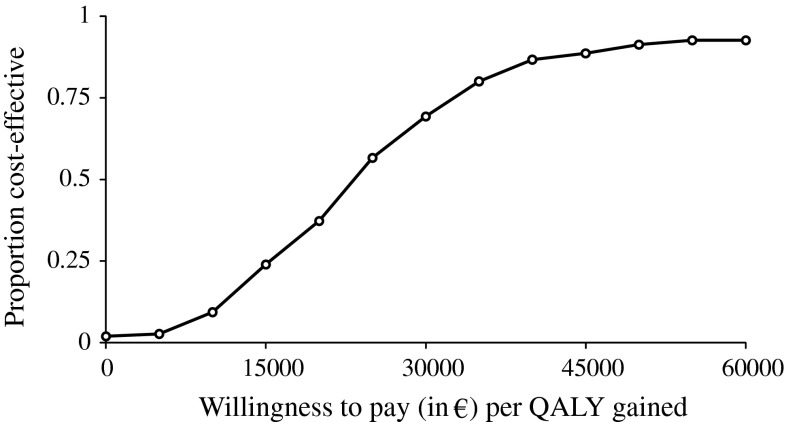
Example of a cost-effectiveness acceptability curve. This graph shows the probability of an osteoporotic treatment being cost-effective compared with no treatment in patients aged 70 years with prevalent vertebral fractures as a function of the decision maker’s willingness-to-pay per one quality-adjusted life-year (*QALY*) [108]. The curve was estimated from probabilistic sensitivity analyses where most parameters (such as therapeutic effect, fracture risk, cost, and disutility) were assigned a probability distribution (e.g., normal or uniform distribution) and values from each distribution were randomly selected during a predefined number of simulations

Models are only as good as their ability to represent the real world. In order for the results and conclusions of economic evaluation to be reliable and valid, it is crucial that the model and the data both represent the reality of the disease as accurately as possible. Guidelines have been developed to increase the quality and reliability of modeling [73, 97]. These include the characterization of uncertainty using appropriate statistical approaches. There could be a substantial amount of uncertainty in the model parameters (and assumptions), and this should be explored using univariate and probabilistic sensitivity analyses. Univariate sensitivity analyses assess the impact of single parameters on the results (which can be represented as a tornado diagram [98]), while probabilistic sensitivity analyses examine the effect of the joint uncertainty surrounding the model variables. Cost-effectiveness acceptability curves can then be constructed to show the probability that the intervention is cost-effective compared with the alternative, for a range of decision makers’ willingness-to-pay thresholds. An example is shown in Fig. 2. Cost-effectiveness acceptability curves have been widely adopted to represent uncertainty in cost-effectiveness analyses [99].

Economic evaluations conducted in the field of osteoporosis are usually based on so-called Markov state-transition models [76]. Markov models are particularly appropriate when a decision problem involves a continuous risk over time, when the timing of events is important, and when events may happen more than once [100], which is the case for osteoporosis. In a Markov model, a cohort of patients is followed over time along mutually exclusive health states (such as healthy, fracture states, and death). At the end of a cycle, patients can move to another health state according to transition probabilities. Values (typically cost and utilities) are assigned to each state, and expected values are then obtained by summing costs and utilities across health states, weighted by the proportion of patients in each state, and then summing across cycles [77]. To assess Markov models, either cohort or individual simulations can be carried out. A microsimulation model follows one individual at a time throughout the model. Due to the probabilistic structure of the model, there will be random variation in individual outcomes (called “first-order uncertainty”) [101], which can be reduced by simulating a large number of patients. The major advantage of microsimulation is that a full patient history is recorded, which increases the reliability of the results and is currently largely compatible with existing state-of-the-art, evidence-based literature [101]. The weakness of such models is that they require more sophisticated and detailed data than cohort-based models. This fact was invoked as a rationale for remaining with cohort modeling approaches in osteoporosis [76].

## Economic Evaluation in Osteoporosis

With limited health-care resources, increasing awareness of osteoporosis, and new diagnostic tools and effective treatments, economic evaluation is increasingly widespread to help decision makers allocate resources in osteoporosis. The number of published economic evaluations in osteoporosis has therefore markedly increased over recent years [76, 102–104]. They have mainly concerned treatment [76, 105, 106] and screening [102, 107] strategies. Recent advances in the diagnosis and treatment of osteoporosis have provided new insights and challenges for economic evaluation, which will be discussed below.

### Economic Evaluation of New Osteoporosis Treatments

As many countries now require economic evaluation as part of the submission file for drug reimbursement, novel drug treatments have been the subject of many economic analyses. Osteoporotic treatments are usually cost-effective in women aged over 60 or 70 years with low bone mass, especially those with prior fractures [76, 104, 105]. In osteoporotic women aged over 80 years, drug therapies are generally reported to be cost-saving [108, 109], meaning that the cost of treating these patients is lower than the averted costs resulting from prevented fractures.

With the development of new products, the question of relevant comparators arises. Health economic evaluations should ideally compare a new intervention with the interventions it is likely to replace. In osteoporosis, there is a lack of head-to-head comparisons, which has led to a paucity of ICER comparisons between active treatments [110]. No treatment (or calcium and vitamin D supplement) appears as the most widely used comparator [76]. Cost-effectiveness analyses often replicate both arms of clinical trials (higher level of evidence) when active treatment is compared with placebo. It has also been argued that the current standard of care is no treatment since osteoporosis is an undertreated disease and the majority of patients with osteoporosis do not receive any treatment [110]. However, this is no longer true since there are many treatments available for osteoporosis that could be considered as standard care. Decision makers are more interested in comparisons between active drugs to determine first-line options. As there is a lack of trial data directly comparing the effectiveness of different treatments, indirect comparison is required to assess cost-effectiveness between active comparators.

Cost-effectiveness analyses between active comparators have started to appear in the osteoporosis literature, for example, for denosumab [98, 111], strontium ranelate [112], and zoledronic acid [113]. Indirect comparisons of efficacy between drugs are less robust because of different baseline characteristics of the populations studied and overlapping confidence intervals for the effect of treatment [114]. Such analyses should therefore be interpreted with great caution.

### Cost-Effective Intervention Thresholds

Recent developments in fracture-risk assessment, such as use of the FRAX algorithm, have led to new applications in health economics of osteoporosis. First, there is a growing body of literature on the interaction between FRAX and treatment efficacy, suggesting that for some agents (e.g., bazedoxifene, clodronate, denosumab) there is a significant interaction between fracture probability and efficacy [115]. This has a significant impact on summary estimates of efficacy and, hence, on cost-effectiveness.

Second, FRAX enables the estimation of risk based on a wider range of clinical risk factors and evaluation of treatment efficacy in populations at differing levels of risk [116]. The cost-effectiveness of drug treatments can therefore be estimated in various types of patients with different combinations of clinical risk factors. FRAX can therefore help to identify new high-risk populations (i.e., patients with different combinations of clinical risk factors) that could benefit from cost-effective treatment.

**Fig. 3:**
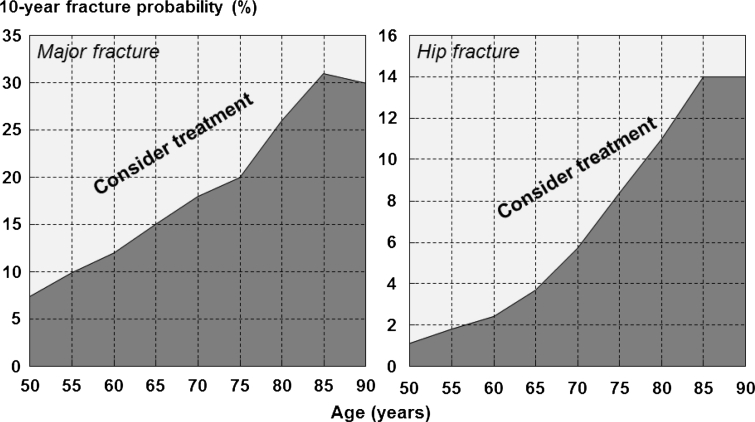
Intervention thresholds in Belgium [119] (copyright permission from Springer)

Finally, economic evaluations are also increasingly being used to determine cost-effective intervention thresholds in order to guide clinical guidelines. Thus, health economic evaluations have been conducted in several countries to determine at what levels of fracture risk treatment should be initiated [80, 81, 117, 118]. In the United Kingdom, the intervention threshold at the age of 50 years corresponds to a 10-year probability of a major osteoporotic fracture of 7.5 % [117]. This increases progressively with age to 30 % at the age of 80 years. In Switzerland, use of a fixed FRAX-based intervention threshold of 15 % for both women and men would permit cost-effective treatment [80]. In Belgium, a “translational approach” was used to define intervention thresholds by examining 10-year fracture probabilities equivalent to those currently accepted for reimbursement of treatment (Fig. 3) [119]. This approach will, however, need to be supported by health economic analyses [119]. Many country-dependent factors could have an impact on intervention thresholds, including fracture cost, intervention cost, and willingness-to-pay [81]. Intervention thresholds should therefore be determined on a per-country basis.

### Economic Value of Improving Adherence

Consideration of new therapeutic options and behavioral interventions that improve medication adherence is currently leading to questions regarding their impact on clinical and economic outcomes. Several studies have assessed the effects of improvements in adherence on fracture outcomes [120–123]. Other studies have estimated the potential economic value (in terms of cost per QALY gained) of interventions that improve medication adherence [36, 37, 64, 124]. Currently, no studies have examined the cost-effectiveness of a specific adherence-enhancing intervention. The economic value of improving adherence was assessed using a variety of hypothetical interventions, which differ according to cost (e.g., marginal or one-time cost) and improvements in adherence (between 10 and 50 %).

**Fig. 4:**
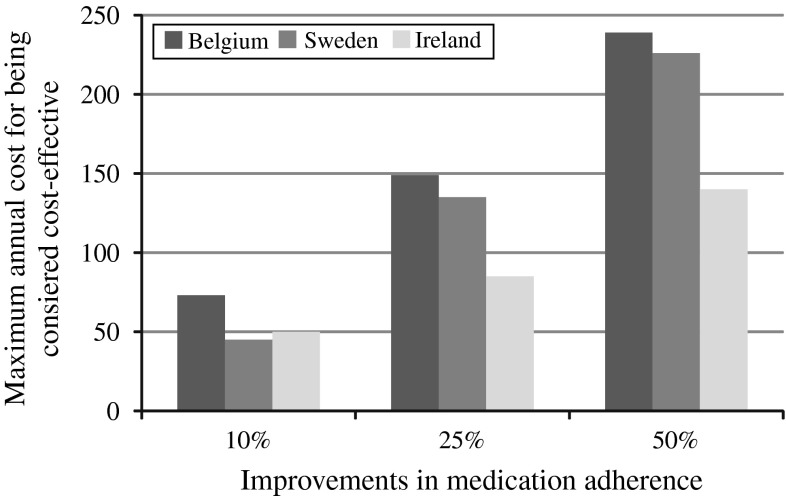
Maximum yearly cost (in euros) for an adherence-enhancing intervention to be considered cost-effective [data from 36, 37, 64]. For Sweden, improvement in medication adherence at 25 % should be read at 30 %. In Ireland, a longer refill gap period (9 weeks) was selected to define persistence resulting in higher base-case adherence levels

The results of these studies suggest that interventions that improve adherence are likely to confer cost-effective benefits [36, 37, 64, 124]. By example, in the United States, a hypothetical intervention with a one-time cost of $250 that reduced discontinuation by 30 % was reported to have an ICER of $29,571 per QALY gained [124]. In studies conducted in Belgium [36], Sweden [37], and Ireland [64], it has been estimated that an intervention that improves adherence by 10 % is cost-effective at a maximum yearly cost of between €45 and €70 (Fig. 4). For a hypothetical intervention that improves adherence by 50 %, it is cost-effective to spend between €140 and €239 per year. The economic value of improving adherence could be situation-specific and improve with the increasing baseline risk for fractures [64, 124].

This work has required methods of incorporating medication adherence into the models. As medication nonadherence affects both costs and outcomes, it could have a substantial impact on the cost-effectiveness of management strategies in osteoporosis and should be incorporated in pharmacoeconomic analyses [64, 122, 125]. In particular, when comparing drugs with different adherence profiles, the lack of inclusion of these concepts could bias the results and lead to suboptimal allocation of resources [126]. Integrating medication adherence into economic analyses in osteoporosis is a complex and difficult task and has been extensively discussed elsewhere [74, 126].

## Discussion

An increasing number of epidemiological and economic studies have revealed the immense burden of osteoporotic fractures, and this is expected to increase further in the future. Information from these studies will help to establish priorities between interventions and diseases and to guide research priorities. Furthermore, economic analyses have suggested that recent advances in the prevention and treatment of osteoporosis, including novel treatments, fracture-risk assessment, and improved medication adherence, are an appropriate and efficient way of allocating health-care resources. Such analyses may also contribute to a more efficient health-care system.

HTA is a rapidly evolving discipline. As more countries use HTA to inform health-care decisions, the harmonization of HTA between jurisdictions has been discussed in order to avoid duplication of effort [127]. Clinical data for new technologies usually apply across countries, but cost-effectiveness (and therefore appraisals of technologies for reimbursement) should be evaluated at the national level because differences in the incidence of the disease, availability of health resources, clinical practice patterns, and relative prices may impact on cost-effectiveness [128]. The development of key principles [129] and good practice, as well as international collaboration between experts, could facilitate a common process for the conduct of HTA for resource-allocation decisions.

There are currently major developments in the methods for economic evaluation in osteoporosis:

- Incorporation of medication adherence into pharmacoeconomic analyses in osteoporosis [74, 126]
- Use of FRAX in the health economics of osteoporosis [116]
- Use of microsimulation models, which are beginning to supplant cohort models in HTA [130]
- In the absence of randomized controlled trials directly comparing active comparators, use of indirect treatment comparisons and network meta-analysis to provide useful evidence for selecting the best option [131]
- Characterization of uncertainty

Alternative approaches to the assessment of QALY have also been developed, including discrete-choice experiments (DCEs) [132, 133] and contingent valuation. DCEs have been increasingly used to elicit collective preferences of subgroups of patients in health care [134]. DCE is an attribute-based survey approach for measuring value, in which patient preference is determined by the levels of different attributes [135]. DCEs help to determine important attributes and provide input on what patients with a particular disease prefer and/or are willing to pay.

Despite the growth of HTA over the past decades, its overall impact on policy making may be limited [14]. The role of science is, however, to inform, not to dictate policy decisions. Humphreys and Piot [136] recently argued that scientific evidence alone is not a sufficient basis for health policy and that other factors (such as democratic and human rights considerations) should be taken into consideration in health policy.

In summary, HTA helps decision makers to efficiently allocate health-care resources. In the field of osteoporosis, HTA reports have revealed a considerable burden of fracture and the economic value of the prevention of fracture and the treatment of osteoporosis.
